# Detection of plant protein in adulterated milk using nontargeted nano‐high‐performance liquid chromatography–tandem mass spectroscopy combined with principal component analysis

**DOI:** 10.1002/fsn3.791

**Published:** 2018-11-20

**Authors:** Jinhui Yang, Nan Zheng, Hélène Soyeurt, Yongxin Yang, Jiaqi Wang

**Affiliations:** ^1^ Ministry of Agriculture – Milk Risk Assessment Laboratory Institute of Animal Science Chinese Academy of Agricultural Sciences Beijing China; ^2^ Ministry of Agriculture – Milk and Dairy Product Inspection Center Beijing China; ^3^ State Key Laboratory of Animal Nutrition Institute of Animal Science Chinese Academy of Agricultural Sciences Beijing China; ^4^ AGROBIOCHEM Department and Teaching and Research Centre (TERRA) Gembloux Agro‐Bio Tech University of Liège Gembloux Belgium; ^5^ Institute of Animal Husbandry and Veterinary Medicine Anhui Academy of Agricultural Sciences Hefei China

**Keywords:** high‐speed centrifugation, milk adulteration, nano‐high‐performance liquid chromatography–tandem mass chromatography, plant protein, sodium dodecyl sulfate–polyacrylamide gel electrophoresis

## Abstract

The objective of this study was to detect plant protein adulterated in fluid milk using nano‐high‐performance liquid chromatography (HPLC)–tandem mass spectroscopy (LC‐MS/MS) combined with proteomics. Unadulterated milk and samples adulterated with soy protein, pea protein, hydrolyzed wheat protein, and hydrolyzed rice protein were prepared, with plant protein level ranged from 0.5% to 8% in total protein. Sodium dodecyl sulfate–polyacrylamide gel electrophoresis (SDS‐PAGE) gels clearly revealed that centrifugation at 20,000 *g* for 60 min would reduce band intensity of casein and albumin in milk. Results of nano‐HPLC‐MS/MS indicated the major proteins of soy (β‐conglycinin, glycinin), pea (vincilin, convicilin, legumin), and wheat (glutenin and gliadin) in adulterated milks, allowing detection of soy protein and hydrolyzed wheat protein at the level above 0.5% in total protein and pea protein at the level of 2 and 4%. No rice protein was identified in milk samples adulterated with hydrolyzed rice protein. Combined with principal component analysis, nano‐HPLC‐MS/MS could discriminate all the adulterated samples from authentic milk. This study demonstrated the feasibility of nano‐HPLC‐MS/MS on the detection of (hydrolyzed) plant protein adulterated in milk.

## 1 INTRODUCTION

Milk products are considered to be the second highest food in the adulteration database, behind olive oil (Moore, Spink, & Lipp, 2012). The addition of foreign nitrogenous compounds to milk products to mask original low protein content is common in dairy adulteration (Nascimento, Santos, Pereira‐Filho, & Rocha, 2017). Adulterants in milk products can cause serious food safety incidents, for example, melamine (Moore et al., 2012). Vegetable protein is a potential candidate to spike milk products for economic reasons (Luykx et al., 2007). Some allergens from plant proteins can cause serious anaphylaxis and disorders (Nakamura & Teshima, 2013), so unlabeled or illegal addition could threaten consumer health and food safety. For these reasons, it is necessary to develop effective techniques to detect plant proteins in milk.

Detection of plant protein in dairy products has been reported in previous literatures. Capillary zone electrophoresis (CZE) has been approved as the official reference method to detect soy protein in skimmed milk powder (Manso, Cattaneo, Barzaghi, Olieman, & Lopez‐Fandino, 2002). An automated fluorescent microsphere‐based flow cytometric triplex immunoassay was developed to detect soy protein (SP), pea protein (PP), and soluble wheat protein in milk powder simultaneously, and the limit of quantification of this triplex immunoassay was above 0.1% (Haasnoot & du Pre, 2007). Detection of soy, pea, wheat, and rice protein at 0.1%–0.2% of sample weight in milk powder was realized by a rapid turbidimetric measure based on the absorbance of the resuspended pellet solution (Scholl, Farris, & Mossoba, 2014), whereas these methods fail to present the origin of these adulterants. With amino acid sequences revealed by fragmented peptides, mass spectrometry (MS) is successful in the identification of plant protein added to milk products (Cordewener et al., 2009; Lu, Liu, Gao, Lv, & Yu, 2017; Luykx et al., 2007). High‐performance liquid chromatography (HPLC)–mass spectrometry (MS) can identify numerous peptides from major seed proteins of soy and pea in the adulterated milk powder, after borate buffer extraction and tryptic digestion (Luykx et al., 2007). Although previous studies have shown that borate buffer was effective to extract insoluble soy and pea protein from milk powder (Luykx et al., 2007; Scholl et al., 2014), the borate buffer enrichment step may not be effective in the detection of plant protein in adulterated fluid milk, because soluble foreign protein is dominant in the adulterated protein and should be the target of detection. Hydrolyzed plant protein tends to have high solubility due to its high content of free amino acids and peptides (Aaslyng et al., 1998). A previous study has found that sodium dodecyl sulfate (SDS)–capillary electrophoresis (CE) failed to detect hydrolyzed SP in adulterated milk powder (Lopez‐Tapia, Garcia‐Risco, Manso, & Lopez‐Fandino, 1999). Combined with multivariable statistics, a variety of nontargeted detection methods have been proposed to identify plant protein adulterated in raw milk. Partial least squares‐discriminant analysis (PLS‐DA) and principal component analysis (PCA) using the fingerprints of intact protein flow injection mass spectra (MS) and ultra‐high‐performance liquid chromatography (UHPLC)–quadrupole time‐of‐flight (QTOF)‐MS were able to detect SP and PP in adulterated milks at the 1% level (Du et al., 2018; Lu et al., 2017). Based on the chromatographic files of authentic and adulterated milk powder obtained by UHPLC with UV detection at 215 nm, the *t* test approach and multivariate Q statistic from a SIMCA model would classify milk powder with SP at 1% and 3% levels correctly and failed to recognize adulterated samples with brown rice and hydrolyzed wheat protein below 10% (Jablonski, Moore, & Harnly, 2014).

The objective of this study was to identify the (hydrolyzed) plant protein in adulterated milk using nontargeted liquid chromatography–tandem mass spectrometry (LC‐MS/MS). PCA is used to reveal the differences in proteins between samples identified by MS. High‐speed centrifugation of samples prior to MS is expected to reduce the cover signal from a high abundance of milk protein over small amounts of adulterant protein, and the corresponding separation would be validated by Sodium dodecyl sulfate–polyacrylamide gel electrophoresis (SDS‐PAGE).

## 2 MATERIALS AND METHODS

### 2.1 Sample preparation

Pasteurized milk samples were purchased from Sanyuan Foods (Beijing, China). The following plant protein products were used in this study: SP isolate (Nature's Bounty, Inc., Bohemia, NY11716, USA), PP isolate (LifeTime Nutritional Specialties, Inc., Orange, CA, USA), HWP (CP100, Conpro, Kangke Food Engineering Tech Ltd., Wuxi, Jiangsu, China), and hydrolyzed rice protein (HRP) (Shuaixing, Yongguodanbaifen Ltd., Wuhan, Hubei, China). About 10 g of plant protein powder was added to 100 ml phosphate buffer (pH = 6.8, 0.2 M). After magnetic stirring overnight, the plant protein solutions were obtained through centrifugation at 5,000 g for 20 min followed by filtration with a 0.2‐μm syringe filter (13 mm, GHP Minispike, Waters). The protein contents in SP, PP, HWP, HRP solution, and milk were 31.0, 23.5, 52.1, 66.4, and 30.7 mg/ml, respectively, as determined by KjelROC analyzer KD310 (OPSIS AB Inc., Sweden) using the Kjeldahl method (IDF 2014).

A series of “adulterated” milks (containing 0.5, 1, 2, 4, and 8 g of plant protein/100 g total protein) were prepared by mixing the plant solution and milk in mass proportions. Skimmed samples were collected after centrifugation at 5,000 g for 20 min. Additional high‐speed centrifugation at 20,000 g for 1 hr was used to prepare samples before further LC‐MS/MS analysis. Both samples (before and after centrifugation at 20,000 g) were analyzed by SDS‐PAGE.

### 2.2 SDS‐PAGE

#### 2.2.1 Gel electrophoresis

Sample protein concentrations were spectrophotometrically determined using bicinchoninic acid (BCA) assay kits (P0010S, Beyotime Institute of Biotechnology, China) before analysis. SDS‐PAGE was undertaken according to Laemmli (1970). After heating at 95°C for 5 min with an equal volume of 2× SDS‐PAGE loading buffer, samples containing 30 mg protein were loaded onto a 12% SDS‐PAGE gel, and the separation was performed at 120 V for 2 hr. The gels were stained for 8 hr in Coomassie blue dye solution [0.12% (w/v) Coomassie brilliant blue G250, 0.12% (w/v) ammonium sulfate, 10% (v/v) phosphoric acid, 20% (v/v) methanol]. This was followed by destaining steps, in which gels were washed by shaking in 10% (v/v) ethanol and 10% (v/v) acetic acid (destaining solution). Triplicate destained gels were scanned and optically analyzed with Quantity One software (V4.6.2, Bio‐Rad, CA, USA). Unique protein bands in the gel of adulterated samples were excised and trypsin digested following the method of Yang et al. (2014).

#### 2.2.2 Protein identification and database search

MS and MS/MS of extracted peptides were collected using a 4800 plus matrix‐assisted laser desorption/ionization (MALDI)–time of flight/time‐of‐flight (TOF/TOF) analyzer (Applied Biosystems, Foster City, CA, USA) equipped with a 355‐nm Nd:YAG laser at an acceleration voltage of 20 kV. Acquisition of positive ions was completed in reflector mode by delayed extraction. Peptide masses ranged from 800 to 4,000 Da. The top eight precursor ions with a signal‐to‐noise ratio of more than 50 for each sample were processed in tandem MS mode with 2500 laser shots and collision energy set as 20 keV. The National Center for Biotechnology Information (NCBI) nonredundant database was used to identify the protein via MASCOT (Matrix Science) search. Peaks with a signal‐to‐noise ratio below 15 were excluded from the search. The search parameters were set as follows: Fixed and variable modifications were carbamidomethylation of cysteine and methionine oxidation, tolerance for one missing cleavage, monoisotopic mass accuracy below 100 ppm and fragment and peptide mass tolerances were ±0.4 Da and ±100 ppm.

### 2.3 LC‐MS/MS analysis

#### 2.3.1 Protein digestion

One hundred microlitre of samples was mixed with same volume of lysis buffer (8 M Urea, 100 mM Tris‐HCl, pH 8.0), treated by ultrasound (100 W, 10 s, interval 15 s, 10 times) and bathed in ice. After centrifugation at 12,000 g at 4°C for 15 min, supernatants were collected for protein concentration test using a Bradford test (Bio‐Rad, Shanghai, China). Then, samples containing 200 μg protein were reduced with dithiothreitol (DTT) at a final concentration of 10 mM and incubated at 37°C for 2 hr. After cooling to room temperature, samples were mixed with 55 mM iodoacetamide, vortexed at 600 rpm for 1 min, and then incubated at 37°C in the dark for 30 min. The same volume of 100 mM NH_4_HCO_3_ was added to samples to decrease urea concentration to less than 2 M. Next, 4 μg trypsin was mixed with the samples and kept at 37°C overnight. The digestion was stopped by addition of 100 μl 60% (v/v) acetonitrile in 0.1% (v/v) formic acid solution. StageTip with Empore C18 extraction disks (3M, South Eagan, MN) was prepared to desalt and dry the samples. Authentic milk (control) and samples adulterated with SP and HWP at 0.5%–4% were prepared in triplicate, and adulteration with PP and HRP at 2% and 4% levels were prepared in duplicate in this part.

#### 2.3.2 LC‐MS/MS analysis

The tryptic digestion products were separated by nano‐HPLC prior to Q Exactive HF Mass Spectrometry (Thermo Scientific). The separation conditions were adapted from Cordewener et al. (2009). Samples were injected on a Thermo Scientific EASY column (C18, 2 cm × 100 μm, 5 μm), which was equilibrated with 95% of solvent A before sample loading, and the peptides were separated on a Thermo Scientific EASY C18 column (100 mm × 75 μm, 3 μm) at a flow rate of 250 nl/min. Solvent A consisted of aqueous 0.1% formic acid solution, and solvent B consisted of 84% acetonitrile in aqueous 0.1% formic acid solution. Gradient conditions started at 5% B, then a linear gradient to 8% B at 2 min, then a linear gradient of 23% B at 90 min, then a linear gradient to 40% B at 105 min, and then a linear gradient to 100% B at 110 min, and 100% B was maintained for the final 10 min.

Peptide analysis was performed in positive ion mode for 120 min, with a selected mass range of 300–1,800 mass/charge (*m/z*). For the survey scan, resolving power was set to 60,000 at *m/z* 200, maximum ion injection time was 50 ms, and the automatic gain control target was 3e6. MS/MS data were acquired using the top 20 most abundant precursor ions, as determined by the survey scan, and activation type was HCD. These were selected with an isolation window of 1.5 *m/z* and fragmented via higher energy collisional dissociation with normalized collision energies of 27 eV. For the MS/MS scans, dynamic exclusion of the selected precursor ions was set to 30 s, resolving power was set to 15,000 at *m/z* 200, and maximum ion injection time was fixed at 50 ms.

### 2.4 Data analysis

Raw files were processed by the MaxQuant software (version 1.5.3.17) of the selected species database. The protein databases of bovine, soybean, pea, wheat, and rice were downloaded from UniProt, which contained 138,035, 250,621, 88,489, 393,298, and 753,301 proteins, respectively. The following parameters were applied: Trypsin was the enzyme, and two missed cleavages were allowed up, and carbamidomethylation of cysteine was defined as a fixed modification; and oxidation of methionine and acetylation of protein N‐term were set as variable modifications. Main search and first search of MS/MS ions were set at 6 and 20 ppm, and MS/MS tolerance was 20 ppm. The false discovery rate for protein and peptide identification was 1%. Relative quantification of identified protein was calculated from the intensities of razor and unique peptides. The decoy database pattern was set as the reverse of the target database.

Identified protein intensities were output to process using Unscrambler software (version 10.4, CAMO AS, Trondheim, Norway). Data processing was described as Cordewener et al. (2009), after log transformation of protein intensities, and data standardization before PCA was performed by centering (subtracting median intensities) and normalization (dividing by the standard deviation).

## 3 RESULTS AND DISCUSSION

### 3.1 SDS‐PAGE

**Figure 1:**
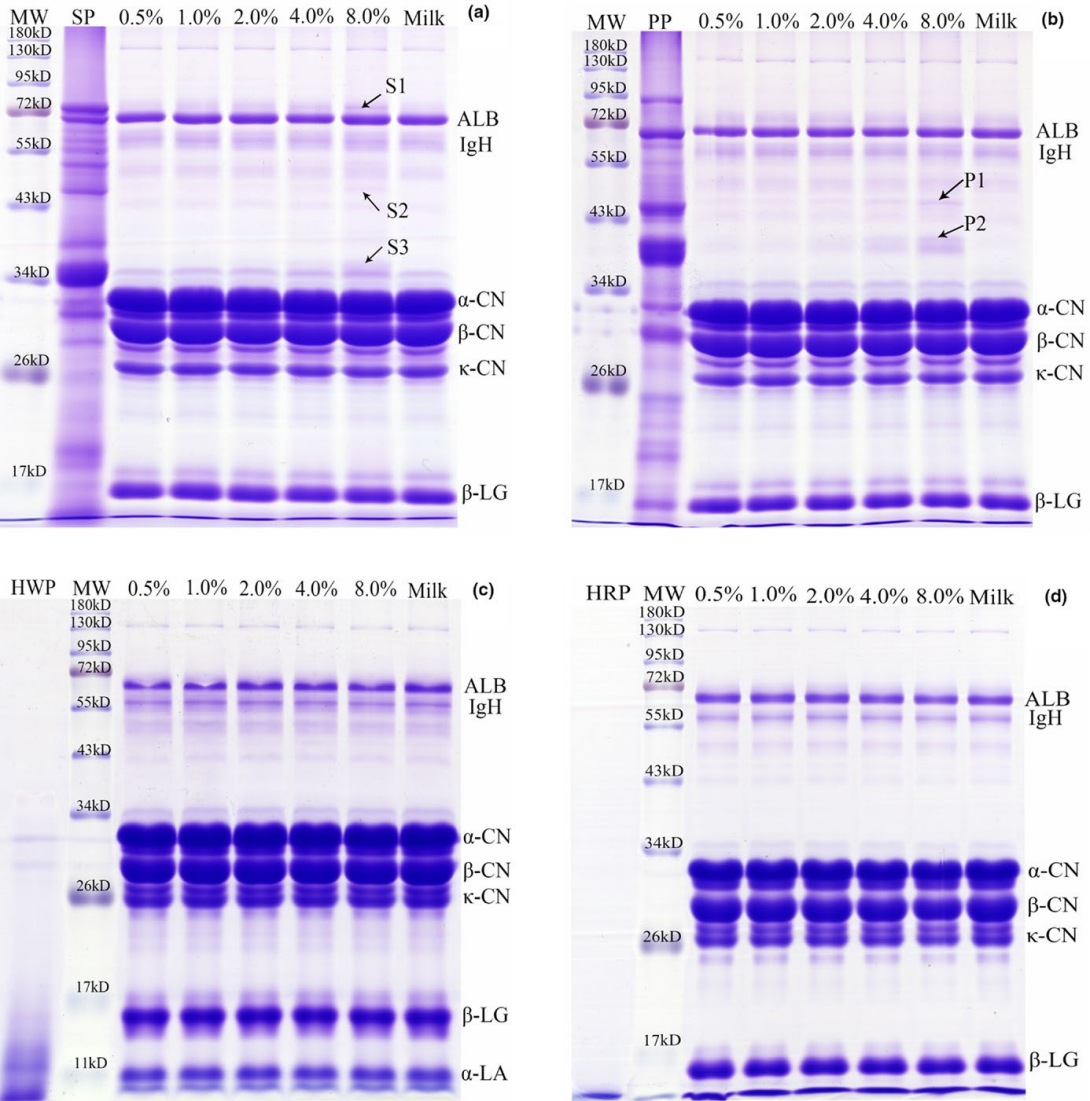
SDS‐PAGE gel profile of milk adulterated with soy protein (a), pea protein (b), hydrolyzed wheat protein (c), and hydrolyzed rice protein (d), and centrifugation at 5,000 g for 20 min. SP: soy protein; PP: pea protein; HWP: hydrolyzed wheat protein; HRP: hydrolyzed rice protein; MW: molecular weight; ALB: albumin; IgH: immunoglobulin heavy chain; CN: casein; α‐LA, α‐lactalbumin; β‐LG: β‐lactoglobulin

**Figure 2:**
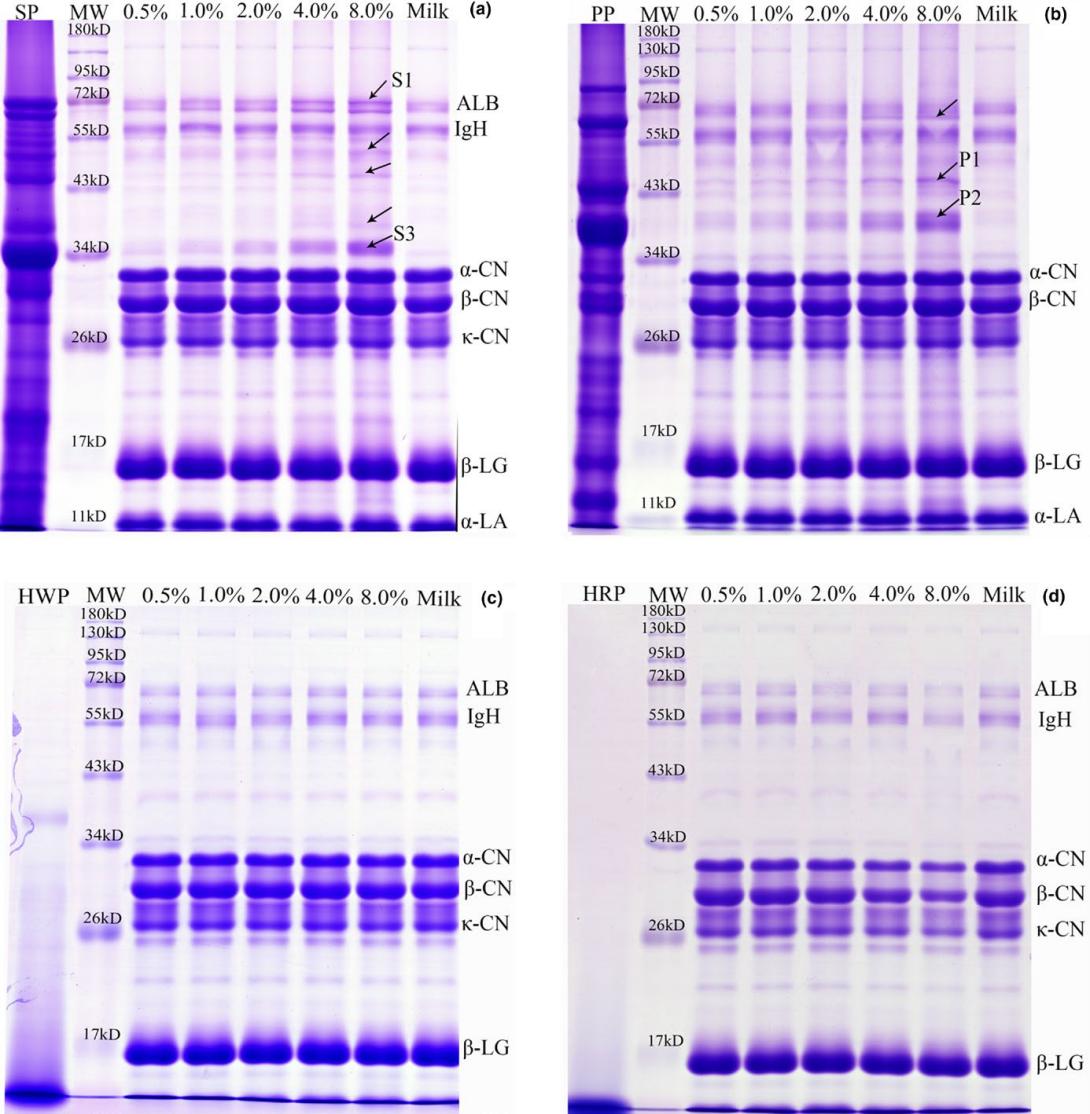
SDS‐PAGE gel profile of milk adulterated with soy protein (a), pea protein (b), hydrolyzed wheat protein (c), and hydrolyzed rice protein (d), and centrifugation at 20,000 g for 60 min. SP: soy protein; PP: pea protein; HWP: hydrolyzed wheat protein; HRP: hydrolyzed rice protein; MW: molecular weight; ALB: albumin; IgH: immunoglobulin heavy chain; CN: casein; α‐LA: α‐lactalbumin; β‐LG: β‐lactoglobulin

Results of SDS‐PAGE of skimmed milk samples and samples treated with high‐speed centrifugation are listed in Figures 1 and 2, respectively. The distinct bands labeled in Figures 1a,b and 2a,b were identified by MALDI‐TOF/TOF MS, and the protein information is listed in Table 1. As shown in Figure 1, the major proteins in skimmed milk consisted of albumin, α‐casein, β‐casein, and κ‐casein, β‐lactoglobulin, and α‐lactalbumin. Several protein bands observed in the lane of SP (Figure 1a) and PP (Figure 1b) had a similar location to SDS‐PAGE data for pea and soy samples reported in a previous study (Scholl et al., 2014), although plant protein extraction methods differed. Although some faint bands are observed between 11 and 17 kDa in the lane of HWP (Figure 1c), most protein fraction residues from HWP and HRP (Figure 1d) are gathered in the bottom line, and this is in line with previous findings, in which 95% of the peptides of hydrolysates were below 1,000 Da (Tessier et al., 2005). Similar protein profiles are presented for milk and adulterated milk with 0.5%–4% levels of SP, and only the 8% level sample shows weak stripes of β‐conglycinin (α and α′ subunit, labeled S2 and S1) and glycinin (G2, labeled S3) besides milk protein (Figure 1a). Obvious stripes of PP (vicilin and legumin A2, labeled P1 and P2) are observed in milk adulteration at 4‐8% level (Figure 1b). No visible lane variance appears between milk and samples adulterated with HWP and HRP (Figure 1c,d).

**Table 1:** Identification of marker protein spots in adulterated milk contrasted with control milk on the gel by MALDI‐TOF MS

Band	ID	Protein name	Organism	Molecular weight (Da)	Protein isoelectric point	Peptide count	Protein Score
P1	P13918	Vicilin	*Pisum sativum*	52,199.7	5.39	25	464
P2	P15838	Legumin A2	*Pisum sativum*	59,233.6	6.21	20	315
P3	Q9M3X6	Convicilin	*Pisum sativum*	72,019.7	5.50	29	374
S1	Q9FZP9	α′ subunit of β‐conglycinin	*Glycine max*	65,103.4	5.23	31	605
S2	Q94LX2	β‐conglycinin α subunit	*Glycine max*	63,248.8	5.00	18	520
S3	A0A0B2PSP9	Glycinin G2	*Glycine soja*	59,013.1	5.79	14	291
S4	O22120	α subunit of β‐conglycinin	*Glycine max*	63,126.9	4.92	20	576
S5	Q9SB12	Glycinin	*Glycine max*	55,337.2	5.46	14	279

After high‐speed centrifugation, weak albumin and casein bands appeared for milk protein (Figure 2), while increased intensity was observed in plant protein lanes. Decreased milk protein intensities indicate more visible foreign protein lines from plant protein in adulterated milk. Additional protein lines are identified as α subunits of β‐conglycinin (S4) and glycinin (S5) emerging at 4 and 8% levels of adulteration with SP. Visible S1, S2, and S3 appeared in adulterated samples at the levels of 1% (Figure 2a). Another protein band (convicilin, labeled P3) from PP could be observed in lanes for 4 and 8% levels of adulteration with PP, and P1 and P2 could be observed at all levels of adulteration with PP (Figure 2b). We found still no obvious difference between different levels of adulteration with HWP after high‐speed centrifugation treatment (Figure 2c). Interestingly, as the adulteration level of HRP increased, the intensities of casein and albumin lines was found to decrease (Figure 2d), possibly as a result of high NaCl content (40% in dry matter) in hydrolyzed plant protein (Aaslyng et al., 1998). Saturation of milk with NaCl precipitates the casein and albumin while the major whey proteins remain soluble (Fox, Uniacke‐Lowe, McSweeney, & O'Mahony, 2015).

Centrifugation at 5,000 g for 20 min was used to prepare skimmed milk in this study, and ultracentrifugation at 100,000 g for 1 hr could sediment most (90%–95%) of the casein from whey (Fox et al., 2015). Therefore, enhanced centrifugation above 5,000 g would reduce the amount of casein in milk, and it is confirmed by the comparison of gel electrophoresis (Figure 1 vs. Figure 2) in the current study. The detection limit of SDS‐PAGE for SP and PP in milk reduced from 8% (soy) and 4% (pea) to 1% and 0.5%, respectively, and more visible SP and PP (S1‐S5, and P1‐P3) lines in adulterated samples appeared after centrifugation at 20,000 g at 4°C for 60 min. In other words, high‐speed centrifugation for skimmed milk is an alternative pretreatment, which may magnify the minor difference between low amounts of plant protein in adulterated milk revealed by successive LC‐MS/MS analysis.

### 3.2 LC‐MS/MS coupled with multivariable statistics

**Figure 3:**
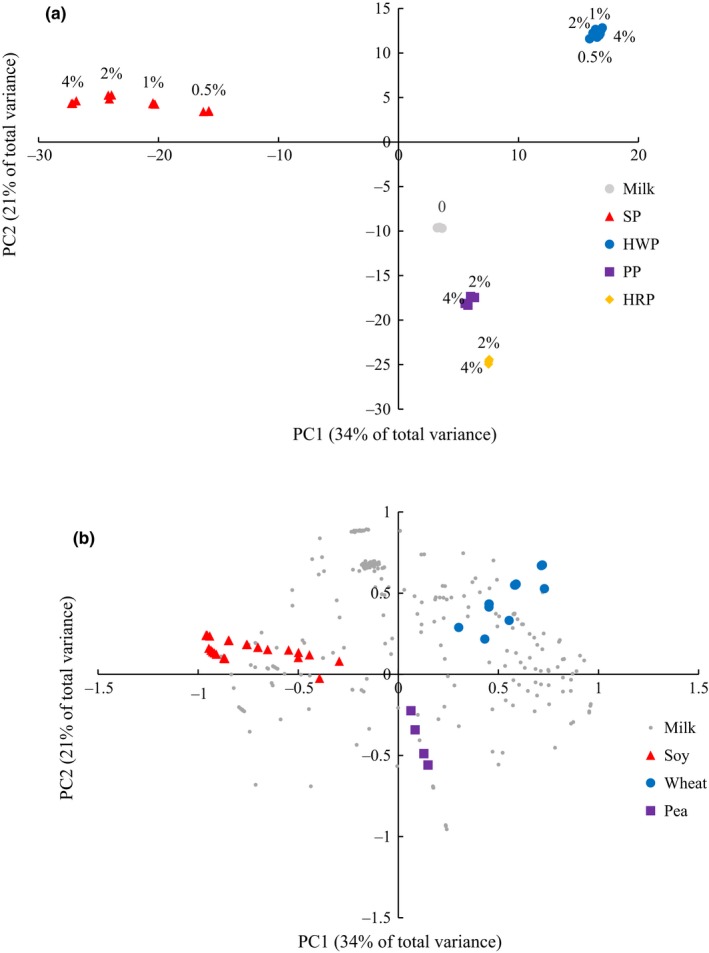
Score (a) and correlation loading (b) plots of principal component analysis (PCA) for adulterated and control milk. a, Numbers labeled above sample points are the percentage of plant protein in total sample milk protein, and different colours indicate the different adulterated (SP, soy protein; PP, pea protein; HWP, hydrolyzed wheat protein; HRP, hydrolyzed rice protein) or control milk samples. Coloured points in (b) show the identified protein from adulterants or milk

The total ion chromatogram of SP, PP, HWP, and HRP is shown in Figure S1. There are 430, 902, 356, and nine proteins identified in SP, PP, HWP, and HRP solutions, respectively. Compared with other plant proteins, fewer peaks appeared in the peptide chromatograms generated from HRP, and fewer proteins were identified. The destruction of tryptophan (Trp) and cysteine (Cys), deamination of glutamine (Gln) and asparagine (Asn), and high levels of hydrolysis occurred in the manufacturing process (Aaslyng et al., 1998) may have disturbed the proteomic identification of rice protein in adulterated milk in this study. More adulterant proteins were identified in milk spiked with HWP than samples with HRP. More peptide peaks observed in the chromatogram profile (Figure 3d) of HWP indicated less extensive hydrolysis in the manufacturing process for wheat protein, a result which was also confirmed by gel electrophoresis (Figure 1c).

Figure S2 shows the summed spectra of replicated measurements for control milk and milk samples adulterated with SP (4% level). No obvious visible difference was observed in the peak intensities of typical LC‐MS runs between replicates or between among samples, which indicates the reproducibility of sample measurements and the similarity of major peptides between samples. Visible differences between pure milk and samples adulterated with SP at 10% level on MS fingerprints, and chromatographic files were observed in recent studies using flow injection MS and UHPLC‐UV detection, respectively (Du et al., 2018; Jablonski et al., 2014). However, direct comparison of chromatograph profiles does not often reveal the difference between adulterated samples and control milk. Discrimination of milk powder adulterated with 5% SP from control samples by visual inspection of peak profiles was not realized in previous reports, using either HPLC‐MS or LC‐QTOF MS (Cordewener et al., 2009; Luykx et al., 2007). Therefore, necessary multivariable statistics, such as PCA, should be used to discover the minor difference in peak profiles between adulterated samples and control milk in this study.

Score and correlation loading plots of PCA analysis are shown in Figure 3. The first two PCs accounted for 56% of the total variance, which clearly distinguishes adulterated milk from control milk. In the loading plot (Figure 3b), identified SP and wheat protein are separated by PC1 for their opposite loading values; PP are also divided for their negative PC2 loadings, while milk protein scatters evenly across the plot. A score plot (Figure 3a) lists each individual LC‐MS/MS profile as one point and replicated sample points overlap. Samples adulterated with different plant proteins cluster into four groups and are separated from authentic milk. Samples adulterated with SP, PP, and HWP tend to have a similar location to corresponding identified adulterant proteins in the loading plot. The distance between each level of adulteration with SP and PP is larger than that of adulteration with HWP and HRP. An approximate linear relationship of data points dependent on protein levels could be observed for samples adulterated with SP. Results of PCA in the current study are similar to those reported in other literature (Cordewener et al., 2009; Lu et al., 2017). Our results show that the adulterated samples could be separated from authentic milk for adulterants proteins. Although no rice protein was identified in the samples adulterated with HRP (Table 2), these samples were also distinguishable from pure milk.

**Table 2:** Summary of samples and identified protein number

Item	Adulterant	Level	Replicates	Number of identified proteins	CV of intensities log values (%)^a^	Percentage of adulterant protein
Milk	None	0	3	418	0.804 (0.02–13.8)	0
Soy 0.5	Soy protein isolate	0.5	3	372	1.077 (0.00–28.5)	23.4
Soy 1	Soy protein isolate	1	3	403	1.121 (0.03–10.8)	29.8
Soy 2	Soy protein isolate	2	3	423	1.052 (0.01–19.5)	33.3
Soy 4	Soy protein isolate	4	3	421	0.809 (0.01–18.1)	37.8
Pea 1	Pea protein isolate	2	2	272	0.831 (0.00–7.00)	20.2
Pea 2	Pea protein isolate	4	2	280	0.672 (0.00–14.6)	21.4
Wheat 0.5	Hydrolyzed wheat protein	0.5	3	329	1.392 (0.01–14.8)	19.8
Wheat 1	Hydrolyzed wheat protein	1	3	333	1.095 (0.06–14.0)	20.7
Wheat 2	Hydrolyzed wheat protein	2	3	337	1.078 (0.06–12.1)	22.8
Wheat 4	Hydrolyzed wheat protein	4	3	339	0.790 (0.00–17.9)	27.4
Rice 2	Hydrolyzed rice protein	2	2	145	0.813 (0.00–6.86)	0
Rice 4	Hydrolyzed rice protein	4	2	145	0.684 (0.00–3.68)	0

a CV, coefficients of variation, expressed as median (range).

Descriptive statistics for proteins in samples identified by LC‐MS/MS are listed in Table 2. Reproducible peak intensities for sample measurements were also presented by coefficients of variation (CV) values, which is ranged from 0.00% to 28.5%, and corresponding medians were below 2%, and this was comparable to previous reports (Cordewener et al., 2009). There were 372–421 and 272–280 proteins identified in samples adulterated with SP and PP, respectively, while 329–339 and 145 proteins were identified in adulteration with HWP and HRP, respectively. About 19.8%–37.8% of the total identified protein was found to be adulterant protein from soy, pea, and wheat, and no rice protein was identified. As the adulterant levels increased, the ratio of identified adulterant protein in total protein also increased, except for adulteration with HRP. The top 10 adulterant proteins from adulteration with SP, PP, and HWP are shown in Table S1. Among them, β‐conglycinin, glycinin, and trypsin inhibitor from SP and vincilin, convicilin, legumin, and provicilin from PP were also identified in other studies, using UHPLC‐QTOF MS proteomics, as reported by Lu et al. (2017). Meanwhile, proteins identified from SDS‐PAGE are also presented in the results of LC‐MS/MS identification. Highly abundant adulterant proteins from HWP derive from gluten proteins in wheat seeds (Garcia‐Molina et al., 2017). In addition, the top 10 most abundant proteins from milk were also defined in our study (Table S2). All these proteins were identified in adulterated milk.

## 4 CONCLUSIONS

In our study, high‐speed centrifugation at 20,000 g for 60 min was found to be an effective pretreatment to reduce highly abundant milk protein in milk samples before MS analysis. LC‐MS/MS protein fingerprints coupled with PCA successfully differentiated adulterated samples (SP and HWP at the level of 0.5%–4%, PP and HRP at the level of 2 and 4%) from authentic milk, and subsequent protein identification allowed the identification of adulterants (SP, PP, and HWP) used in milk adulteration. However, no rice protein was identified in the samples adulterated with HRP. The identification of adulterants protein by LC‐MS/MS may be disturbed by the degree of hydrolysis of plant protein.

## CONFLICT OF INTEREST

The authors declare that they have no conflict of interest.

## ETHICAL STATEMENT

There is no conflict of interest to declare. This article does not contain any studies with human participants or animals.

## Supporting information


** **

** **

** **
