# Supplementary material for: Detection of plant protein in adulterated milk using nontargeted nano‐high‐performance liquid chromatography–tandem mass spectroscopy combined with principal component analysis
Source: Food Sci Nutr. 2018 Nov 20;7(1):56–64. doi: 10.1002/fsn3.791 (PMC6341172; doi:10.1002/fsn3.791)
Supplement: Supplementary file 1 [file FSN3-7-56-s001.pdf]

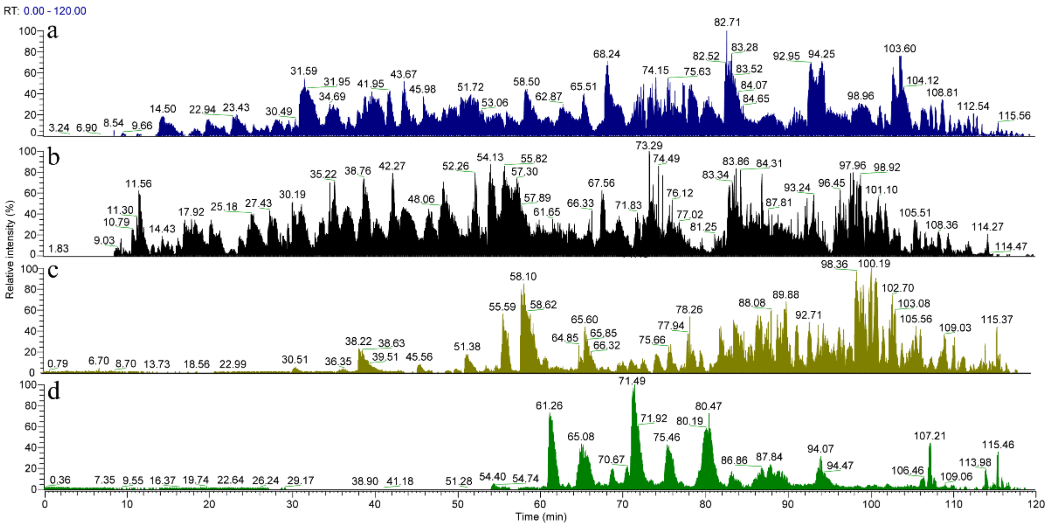

Supplementary Figure S1. Base peak chromatogram of mass spectra of soy protein solution (a), pea protein solution (b), hydrolysed wheat protein solution (c), and hydrolysed rice protein solution (d)
