# Supplementary material for: Detection of plant protein in adulterated milk using nontargeted nano‐high‐performance liquid chromatography–tandem mass spectroscopy combined with principal component analysis
Source: Food Sci Nutr. 2018 Nov 20;7(1):56–64. doi: 10.1002/fsn3.791 (PMC6341172; doi:10.1002/fsn3.791)
Supplement: Supplementary file 2 [file FSN3-7-56-s002.pdf]

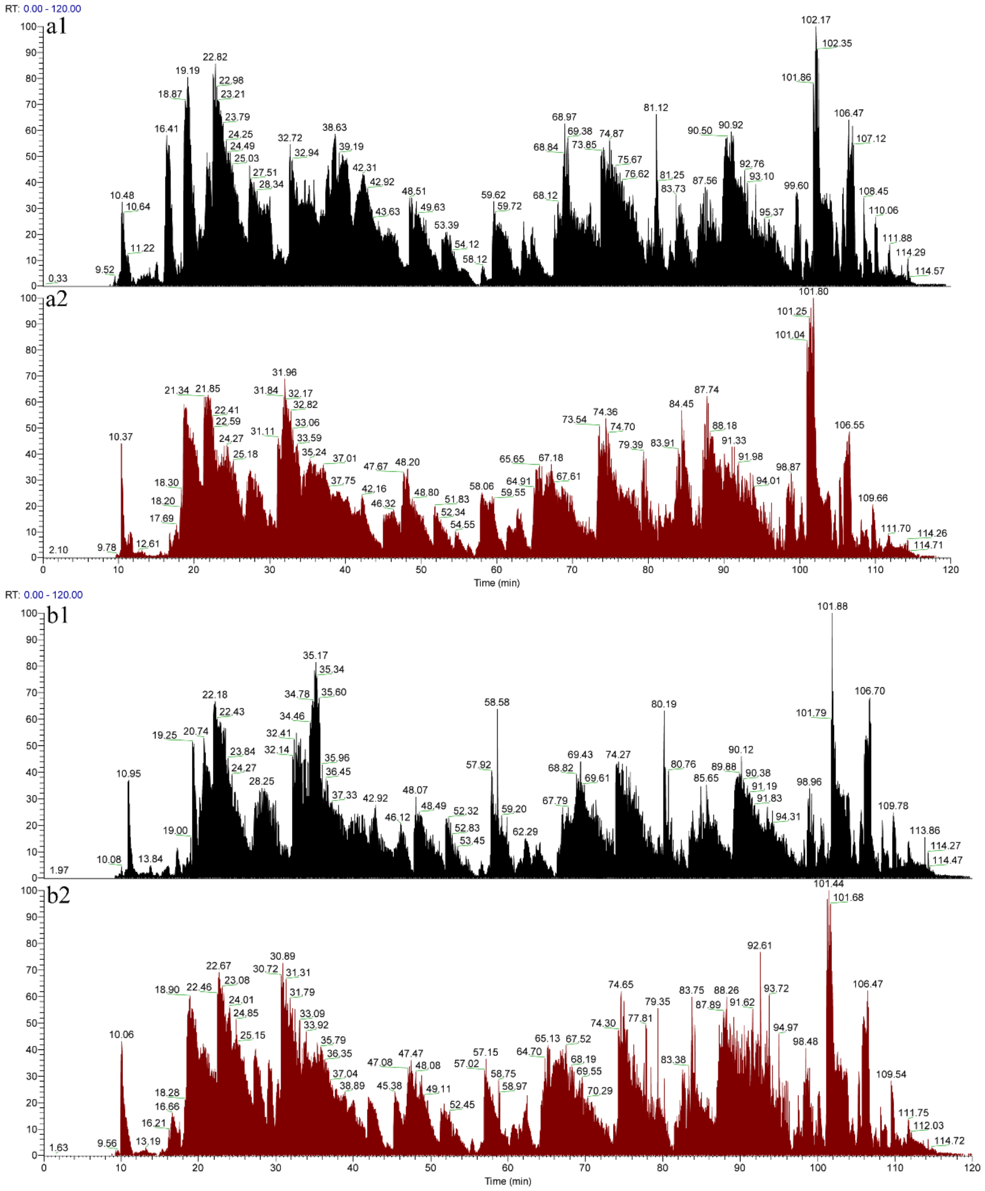

Supplementary Figure S2. Base peak chromatogram of mass spectra of milk (a1, a2) and adulterated samples (b1, b2) with soy protein at 4% of total protein
