# Supplementary material for: Detection of plant protein in adulterated milk using nontargeted nano‐high‐performance liquid chromatography–tandem mass spectroscopy combined with principal component analysis
Source: Food Sci Nutr. 2018 Nov 20;7(1):56–64. doi: 10.1002/fsn3.791 (PMC6341172; doi:10.1002/fsn3.791)
Supplement: Supplementary file 3 [file FSN3-7-56-s003.docx]

**Supplementary information**

**Detection of plant protein in adulterated milk using non-targeted liquid chromatography -tandem mass spectroscopy combined with principal component analysis**

Jinhui Yang^1,2,3,4^, Nan Zheng^1,2,3^*, Hélène Soyeurt^4^, Yongxin Yang^5^, Jiaqi Wang^1,2,3^

^1^ Ministry of Agriculture – Milk Risk Assessment Laboratory, Institute of Animal Science, Chinese Academy of Agricultural Sciences, Beijing 100193, China;

^2^ Ministry of Agriculture – Milk and Dairy Product Inspection Center, Beijing 100193, China;

^3^ State Key Laboratory of Animal Nutrition, Institute of Animal Science, Chinese Academy of Agricultural Sciences, Beijing 100193, China;

^4^ AGROBIOCHEM Department and Teaching and Research Centre (TERRA), Gembloux Agro-Bio Tech, University of Liège, 5030 Gembloux, Belgium;

^5^ Institute of Animal Husbandry and Veterinary Medicine, Anhui Academy of Agricultural Sciences, Hefei 230031, China

Corresponding author: Nan Zheng

Email: zhengnan_1980@126.com

Tel: 86-10-62816069

Fax: 86-10-62816069

**Table S1.** Top 10 proteins identified from each adulterant in corresponding adulterated milk

| Adulterants | Protein name | Organism | Protein numbers | Razor + unique peptides | Mass (kDa) | Coverage (%) | Score |
| --- | --- | --- | --- | --- | --- | --- | --- |
| Soy protein | Alpha' subunit of β-conglycinin | *Glycine max* | 9 | 43 | 65.14 | 59.2 | 323.31 |
|  | Alpha subunit of β-conglycinin | *Glycine max* | 10 | 33 | 63.16 | 63.5 | 323.31 |
|  | Glycinin A3B4 subunit | *Glycine soja* | 13 | 19 | 57.70 | 70.6 | 323.31 |
|  | Glycinin | *Glycine max* | 6 | 32 | 63.80 | 62.7 | 323.31 |
|  | Uncharacterised protein | *Glycine max* | 5 | 7 | 18.46 | 43.7 | 221.18 |
|  | Kunitz trypsin inhibitor | *Glycine max* | 23 | 13 | 24.14 | 44.9 | 311.72 |
|  | Uncharacterised protein | *Glycine max* | 3 | 25 | 57.99 | 65.1 | 323.31 |
|  | Beta-conglycinin β subunit | *Glycine max* | 1 | 24 | 48.33 | 67.1 | 323.31 |
|  | Seed maturation protein PM31 | *Glycine max* | 2 | 11 | 17.75 | 52.9 | 248.57 |
|  | Uncharacterised protein | *Glycine max* | 1 | 27 | 54.68 | 72.5 | 323.31 |
| Pea protein | Convicilin | *Pisum sativum* | 1 | 34 | 72.06 | 65.9 | 323.31 |
|  | Vicilin | *Pisum sativum* | 4 | 24 | 64.6 | 47.3 | 323.31 |
|  | Legumin A2 | *Pisum sativum* | 2 | 32 | 59.27 | 61.9 | 323.31 |
|  | Provicilin | *Pisum sativum* | 2 | 12 | 31.54 | 70.5 | 323.31 |
|  | Vicilin 47k | *Pisum sativum* | 1 | 3 | 49.66 | 72.1 | 255.19 |
|  | P54 protein | *Pisum sativum* | 1 | 28 | 54.66 | 61.5 | 323.31 |
|  | Legumin (Minor small) | *Pisum sativum* | 1 | 20 | 64.87 | 55.1 | 323.31 |
|  | LegA class | *Pisum sativum* | 3 | 10 | 58.79 | 61.9 | 323.31 |
|  | Vicilin, 14 kDa component | *Pisum sativum* | 1 | 2 | 14.04 | 51.6 | 89.15 |
|  | Legumin J | *Pisum sativum* | 1 | 13 | 56.90 | 69.2 | 323.31 |
| Hydrolysed wheat protein | Alpha-amylase inhibitor CM3 | *Triticum turgidum subsp. Durum* | 3 | 8 | 18.22 | 75.6 | 323.31 |
|  | 0.19 dimeric α-amylase inhibitor | *Triticum aestivum* | 25 | 5 | 13.34 | 91.1 | 323.31 |
|  | 0.19 dimeric α-amylase inhibitor | *Triticum aestivum* | 8 | 8 | 13.25 | 89.5 | 323.31 |
|  | Low molecular weight glutenin subunit | *Triticum aestivum* | 95 | 3 | 34.78 | 18.5 | 323.31 |
|  | Gamma-gliadin | *Triticum dicoccoides* | 1 | 2 | 14.6 | 29.2 | 94.17 |
|  | Alpha-amylase/trypsin inhibitor CM3 | Triticum aestivum | 1 | 2 | 17.30 | 52.5 | 19.22 |
|  | High molecular weight glutenin subunit Bx17 | Triticum aestivum | 43 | 6 | 80.07 | 12.6 | 100.22 |
|  | Uncharacterised protein | Triticum aestivum | 10 | 5 | 21.89 | 35.8 | 123.43 |
|  | Alpha-amylase inhibitor CM1 | Triticum aestivum | 4 | 5 | 13.10 | 68.3 | 323.31 |
|  | Putative α-amylase inhibitor CM2 | Triticum aestivum | 1 | 4 | 13.01 | 60.0 | 321.18 |

**Table S2.** Top 10 identified milk proteins in unadulterated (control) milk

| Protein name | Organism | Number of proteins | Razor + unique peptides | Mass (kD) | Coverage (%) | Score |
| --- | --- | --- | --- | --- | --- | --- |
| Beta-lactoglobulin | Bos taurus | 7 | 24 | 19.97 | 86.5 | 323.31 |
| Kappa-casein | Bos indicus | 20 | 11 | 17.33 | 58.3 | 323.31 |
| Alpha-S1-casein | Bos taurus | 3 | 17 | 24.53 | 59.3 | 323.31 |
| Serum albumin | Bos taurus | 5 | 58 | 69.32 | 74.5 | 323.31 |
| Alpha-lactalbumin | Bos taurus | 7 | 18 | 14.16 | 65.9 | 323.31 |
| Lactoferrin | Bos taurus | 14 | 47 | 76.27 | 67.1 | 323.31 |
| Lactadherin | Bos taurus | 5 | 26 | 47.41 | 58.3 | 323.31 |
| Beta-lactoglobulin | Bos taurus | 2 | 4 | 19.88 | 86.5 | 323.31 |
| Beta-casein | Bos taurus | 2 | 5 | 25.00 | 25.0 | 187.74 |
| Polymeric immunoglobulin receptor | Bos taurus | 2 | 30 | 82.43 | 47.3 | 323.31 |
